# Evaluation of Long-Term Response to Biological Therapy in Severe Asthma and Considerations for Treatment Adjustment

**DOI:** 10.3390/jcm14082623

**Published:** 2025-04-11

**Authors:** Jordan Giordani, Alessandro Pini, Laura Pini

**Affiliations:** 1 Department of Clinical and Experimental Sciences, University of Brescia, 25123 Brescia, Italy; 2 Department of Emergency, Anaesthesiological and Resuscitation Sciences, University Cattolica Sacro Cuore, 00168 Rome, Italy; 3 ASST Spedali Civili di Brescia, 25123 Brescia, Italy

Assessing long-term responses to biological therapies for severe asthma is critical for optimizing patient management and improving clinical outcomes. The literature provides a significant amount of data from long-term and real-life studies, which are essential for evaluating the durability, efficacy, and impact of these treatments over time. Long-term studies focus on the sustained effects of biological agents, while real-world studies offer insights into their effectiveness in everyday clinical settings, enhancing our understanding of treatment impacts on patient populations [1].

Recent advancements in the airway remodeling field have further refined the evaluation of biological therapies. Notably, Omalizumab, an anti-IgE monoclonal antibody, has been shown to inhibit IgE-mediated extracellular matrix (ECM) deposition and reduce reticular basement membrane thickness and fibronectin deposition [2]. Similarly, Mepolizumab, an anti-IL-5 agent, has improved airway remodeling by reducing eosinophil counts and tenascin expression [3]. Benralizumab, an anti-IL-5R agent, has been observed to diminish eosinophil counts associated with reductions in airway smooth muscle mass [4]. Dupilumab, an IL-4 receptor antagonist, has shown promise in preventing eosinophil infiltration into lung tissue, with ongoing studies evaluating its impact on lung function and structure [5]. At the same time, Tezepelumab, targeting thymic stromal lymphopoietin (TSLP), has demonstrated reductions in airway inflammation and remodeling in animal models, although human studies are still in progress [6].

An important recent therapeutic development is Depemokimab, a long-acting monoclonal antibody targeting IL-5. The phase III clinical trials SWIFT-1 and SWIFT-2 demonstrated that biannual Depemokimab administration led to a 54% reduction in exacerbations compared to placebo, along with the sustained suppression of type 2 inflammation, and improved the quality of life in patients with severe eosinophilic asthma [7].

In addition, more recent studies have investigated the use of Benralizumab during acute phases of severe asthma. A study published in *The Lancet Respiratory Medicine* showed that a single Benralizumab injection during an exacerbation reduced the need for additional treatments by 30% compared to oral steroids, with fewer side effects and the potential for at-home administration [8].

The long-term response to biological therapies can also be evaluated through their impact on the natural history of severe asthma. Patients who respond positively to treatment may experience two distinct outcomes upon discontinuation: a recurrence of symptoms or sustained remission. The XPORT study, a multicenter randomized controlled trial, reported that 33% of patients who discontinued Omalizumab experienced exacerbations compared to 52% in the placebo group, indicating a potential for sustained disease control [9]. Furthermore, a prospective study by Vennera et al. found that 60% of patients maintained long-term benefits up to four years after Omalizumab discontinuation [10]. Conversely, Haldar et al. reported an increase in exacerbations related to eosinophilia in patients who discontinued Mepolizumab after 12 months [11]. However, the COSMO study indicated that a 12-week Mepolizumab discontinuation did not significantly affect asthma control, as measured by the Asthma Control Questionnaire (ACQ5) [12]. These findings underline the variability in patient responses after biological therapy discontinuation.

Identifying candidates for biological therapy discontinuation necessitates a nuanced approach. When considering treatment adjustment or discontinuation, patients can be categorized into super-responders, partial responders, and non-responders. Super-responders may exhibit minimal residual symptoms, stable lung function without the need for oral corticosteroids (OCSs), and low fractional exhaled nitric oxide (FeNO) and eosinophil levels during treatment [13,14]. In contrast, partial and non-responders may require a reassessment of their treatment regimen. Real-world data from the ISAR registry and the CHRONICLE study indicated that approximately 79% of patients continued their current biologic therapy, while 10% discontinued and 11% switched to another biologic [15]. The decision to switch is often prompted by inadequate clinical response, adverse events, or patient preferences regarding treatment administration [16].

The assessment of response to biological therapy should be based on multiple outcomes: symptom improvement, rescue medication use, lung function, reduction in OCS, quality of life, and healthcare resource utilization [17]. A patient may be classified as a non-responder after 4–6 months of treatment if there is minimal clinical improvement, no steroid use reduction, and persistent eosinophilia or T2-low inflammation. Factors contributing to non-response may include disease mechanisms not targeted by the chosen biologic, comorbidities, poor adherence, or the presence of drug-neutralizing antibodies [18].

Currently, there are no specific guidelines on the timing and criteria for switching between biologics, but it is generally recommended that treatment is reassessed after 4–12 months. In many cases, switching directly from one biologic to another (e.g., from Omalizumab to Mepolizumab) has been shown to be safe, even without a wash-out period [19,20].

In conclusion, the long-term response to biological therapies has revolutionized the management of severe asthma, improving long-term outcomes and offering personalized treatment strategies. The Special Issue “Clinical Advances in Allergy and Asthma: Issues, Strategies, and Future Directions” primarily aims to provide innovative insights and increase readers’ awareness of the new frontiers gradually opening up in treating severe asthma. In this context, biologic therapies are the most promising and clinically effective vanguard. The advances achieved so far and the possibility of tailored diagnostic and therapeutic approaches open up the possibility of leading patients toward the ambitious goal of clinical remission.

## References

[1] Brusselle G.G., Koppelman G.H. (2022). Biologic Therapies for Severe Asthma. N. Engl. J. Med..

[2] Domingo C., Mirapeix R.M., González-Barcala F.J., Forné C., García F. (2023). Omalizumab in Severe Asthma: Effect on Oral Corticosteroid Exposure and Remodeling. A Randomized Open-Label Parallel Study. Drugs.

[3] Varricchi G., Poto R., Lommatzsch M., Brusselle G., Braido F., Virchow J.C., Canonica G.W. (2025). Biologics and airway remodeling in asthma: Early, late, and potential preventive effects. Allergy.

[4] Visca D., Ardesi F., Zappa M., Grossi S., Pignatti P., Vanetti M., Pini L., Sotgiu G., Centis R., Migliori G.B. (2024). The effect of benralizumab on inflammation in severe asthma: A real-life analysis. Ther. Adv. Respir. Dis..

[5] Tajiri T., Suzuki M., Nishiyama H., Ozawa Y., Kurokawa R., Takeda N., Ito K., Fukumitsu K., Kanemitsu Y., Mori Y. (2024). Efficacy of dupilumab for airway hypersecretion and airway wall thickening in patients with moderate-to-severe asthma: A prospective, observational study. Allergol Int..

[6] Diver S., Khalfaoui L., Emson C., Wenzel S.E., Menzies-Gow A., Wechsler M.E., Johnston J., Molfino N., Parnes J.R., Megally A. (2021). Effect of tezepelumab on airway inflammatory cells, remodelling, and hyperresponsiveness in patients with moderate-to-severe uncontrolled asthma (CASCADE): A double-blind, randomised, placebo-controlled, phase 2 trial. Lancet Respir. Med..

[7] Jackson D.J., Wechsler M.E., Jackson D.J., Bernstein D., Korn S., Pfeffer P.E., Chen R., Saito J., de Luíz Martinez G., Dymek L. (2024). SWIFT-1 and SWIFT-2 Investigators, SWIFT-1 Investigators, & SWIFT-2 Investigators. Twice-Yearly Depemokimab in Severe Asthma with an Eosinophilic Phenotype. N. Engl. J. Med..

[8] Ramakrishnan S., Russell R.E.K., Mahmood H.R., Krassowska K., Melhorn J., Mwasuku C., Pavord I.D., Bermejo-Sanchez L., Howell I., Mahdi M. (2025). Treating eosinophilic exacerbations of asthma and COPD with benralizumab (ABRA): A double-blind, double-dummy, active placebo-controlled randomised trial. Lancet Respir. Med..

[9] Ledford D., Busse W., Trzaskoma B., Omachi T.A., Rosén K., Chipps B.E., Luskin A.T., Solari P.G. (2017). A randomized multicenter study evaluating Xolair persistence of response after long-term therapy. J. Allergy Clin. Immunol..

[10] Vennera M.D.C., Sabadell C., Picado C., Spanish Omalizumab Registry (2018). Duration of the efficacy of Omalizumab after treatment discontinuation in ‘real life’ severe asthma. Thorax.

[11] Haldar P., Brightling C.E., Singapuri A., Hargadon B., Gupta S., Monteiro W., Bradding P., Green R.H., Wardlaw A.J., Ortega H. (2014). Outcomes after cessation of mepolizumab therapy in severe eosinophilic asthma: A 12-month follow-up analysis. J. Allergy Clin. Immunol..

[12] Ortega H., Lemiere C., Llanos J.P., Forshag M., Price R., Albers F., Yancey S., Castro M. (2019). Outcomes following mepolizumab treatment discontinuation: Real-world experience from an open-label trial. Allergy Asthma Clin. Immunol..

[13] Moore W.C., Kornmann O., Humbert M., Poirier C., Bel E.H., Kaneko N., Smith S.G., Martin N., Gilson M.J., Price R.G. (2022). Stopping versus continuing long-term mepolizumab treatment in severe eosinophilic asthma (COMET study). Eur. Respir. J..

[14] Jeffery M.M., Inselman J.W., Maddux J.T., Lam R.W., Shah N.D., Rank M.A. (2021). Asthma Patients Who Stop Asthma Biologics Have a Similar Risk of Asthma Exacerbations as Those Who Continue Asthma Biologics. J. Allergy Clin. Immunol. Pract..

[15] Menzies-Gow A.N., McBrien C., Unni B., Porsbjerg C.M., Al-Ahmad M., Ambrose C.S., Dahl Assing K., von Bülow A., Busby J., Cosio B.G. (2022). Real World Biologic Use and Switch Patterns in Severe Asthma: Data from the International Severe Asthma Registry and the US CHRONICLE Study. J. Asthma Allergy.

[16] Nagase H., Suzukawa M., Oishi K., Matsunaga K. (2023). Biologics for severe asthma: The real-world evidence, effectiveness of switching, and prediction factors for the efficacy. Allergol. Int..

[17] Roberts G. (2020). Understanding the response to asthma biological therapy. Clin. Exp. Allergy.

[18] Saco T., Ugalde I.C., Cardet J.C., Casale T.B. (2021). Strategies for choosing a biologic for your patient with allergy or asthma. Ann. Allergy Asthma Immunol..

[19] Canonica G.W., Bagnasco D., Bondi B., Varricchi G., Paoletti G., Blasi F., Paggiaro P., Braido F., SANI Study Group (2024). SANI clinical remission definition: A useful tool in severe asthma management. J. Asthma.

[20] Chapman K.R., Albers F.C., Chipps B., Muñoz X., Devouassoux G., Bergna M., Galkin D., Azmi J., Mouneimne D., Price R.G. (2019). The clinical benefit of Mepolizumab replacing Omalizumab in uncontrolled severe eosinophilic asthma. Allergy.

